# Pre-Residency Research Engagement and Subsequent Publication Output Among Orthopaedic Surgery Applicants

**DOI:** 10.2106/JBJS.OA.26.00158

**Published:** 2026-07-13

**Authors:** Ben Setaro, Evan London, Siddarth Dasari, Dominic M. Farronato, A. Bobby Chhabra, Wendy Novicoff, Charles A. Su

**Affiliations:** 1 School of Medicine, University of Virginia, Charlottesville, Virginia; 2 Department of Orthopaedic Surgery, University of Virginia, Charlottesville, Virginia

## Abstract

**Introduction:**

Competition for orthopaedic surgery residency positions has intensified in recent years, coinciding with the shift of the United States Medical Licensing Examination Step 1 and many medical schools’ curricula to pass/fail grading. Consequently, residency programs have placed greater emphasis on applicants’ research productivity. The average number of research items listed on applications has quadrupled since 2011. However, it is unclear whether preresidency research productivity predicts long-term research output. This study evaluates the association between preresidency research productivity and future publication output.

**Methods:**

A retrospective cohort of 822 applicants to a single US orthopaedic surgery residency program during the 2010 application cycle was evaluated. Electronic Residency Application Service (ERAS) data collected included the number and total hours of research experiences, total research items, oral/poster presentations, and manuscript publications. Each publication was cross-referenced using Scopus and PubMed to determine citation counts and publication in a top-15 orthopaedic journal. Applicants were matched to publicly available Scopus profiles to assess post-ERAS submission research productivity. The primary outcome was the number of post-ERAS publications. The 309 applicants without Scopus records were excluded from the study. Associations between variables were analyzed using Spearman’s correlation, with statistical significance set at p < 0.05.

**Results:**

Post-ERAS publication count was significantly correlated with the number of poster/oral presentations (P = 0.023) and publication in a top-15 orthopaedic journal (P = 0.027). However, post-ERAS publication count was not significantly associated with the number of research experiences (P = 0.129), research hours (P = 0.501), total research items (P = 0.571), manuscript count (P = 0.713), or cumulative citations from application manuscripts (P = 0.308).

**Discussion:**

Poster/oral presentations and publication in a top-15 orthopaedic journal were the only ERAS metrics associated with postapplication publication output. Total research items, manuscripts, and citations were not correlated with continued productivity. Qualitative indicators of research engagement, such as presentations and publication quality, were associated with continued publication output, whereas quantitative metrics were not. These findings suggest that meaningful research involvement, rather than volume alone, better predicts long-term publication output.

**Level of Evidence:**

Level III. See Instructions for Authors for a complete description of levels of evidence.

## Introduction

Matching into orthopaedic surgery is highly competitive, with about 1 in every 5 applicants annually failing to match^1^. The shift of the United States Medical Licensing Examination Step 1 and many medical schools' curricula to pass/fail grading amplified these pressures by altering the weight placed on other objective metrics. National analyses show rising research productivity and declining work and volunteer experiences among applicants after the grading changes—patterns that may reflect heightened concerns about research expectations or expanding use of research years^2^. Forecasting models project that by 2040, matched applicants will average 165.6 research items and a Step 2 score of 266^3^.

These unsustainable increases parallel historical trends, even before Step 1 became pass/fail. Between 2007 and 2014, match rates remained at approximately 80%, yet applicants’ Step scores increased and research activity doubled^4,5^. To meet expectations, applicants bolster their applications with numerous research projects. Nearly half of orthopaedic surgery applicants list unpublished manuscripts, fewer than two-thirds of which reach publication^6^.

Evidence examining the downstream impact of early research activity shows mixed patterns. Within orthopaedics, applicants with 2 or more publications before residency tend to maintain greater research productivity throughout residency, independent of program reputation^7^. Greater research output during residency also correlates with higher productivity as a physician. However, findings from other fields challenge the predictive value of preresidency publication volume^8^. A study of neurosurgery applicants found no link between preresidency publication counts and research output during residency^9^. Additional work in orthopaedics revealed that research fellowships and PhDs are associated with higher h-indices later in one’s career^10^.

The mixed picture painted by previous literature highlights an important gap in understanding how different forms of preresidency research translate into long-term publication output within orthopaedic surgery. Given the evolving landscape with pass/fail scoring, increasing applicant engagement in research, and rising program expectations, it remains unclear which components of an applicant’s Electronic Residency Application Service (ERAS) research record have the strongest association with future research output. Moreover, research quantity is increasingly emphasized during residency selection, both at expense of other components such as personal statements and recommendation letters that may better elucidate personal qualities and without consistent data showing association between research quantity and future output. Therefore, clarifying these relationships is essential for applicants striving to build meaningful research portfolios and programs seeking metrics that reflect future potential. The purpose of this study was to evaluate how distinct ERAS research components relate to future manuscript output 10 years into practice.

## Materials and Methods

Following institutional review board approval, a retrospective cohort analysis of all 822 applicants to a single US orthopaedic surgery residency program during the 2010 ERAS cycle was performed. This residency program is at an academic medical center, has 25 residents, and is ranked in the top 40^11^. ERAS data were shared by the Association of American Medical Colleges under licensing agreement. The number of extracurricular experiences was totaled for each applicant. Applicants had been previously instructed to apply ERAS categories of Research, Volunteer, or Work to each experience. A count of experiences for each category and time per experience was generated.

Total research item count and number of research items by category were retrieved for each applicant. Research items were initially tallied by 9 ERAS-defined labels: peer-reviewed journal articles/abstracts, journal articles/abstracts (other-than-published), peer-reviewed book chapters, scientific monographs, other articles, poster presentations, oral presentations, peer-reviewed online publications, and non–peer-reviewed online publications. Of these, poster presentations and oral presentations were combined into a single metric. Similarly, journal articles/abstracts (other-than-published) denoted as ‘accepted’ or ‘in press’ were combined with peer-reviewed count to generate a single abstract/article count.

To quantify abstracts versus manuscripts, each publication was assessed by title using the Scopus search engine. Journal of publication was recorded to determine if applicants had published in a top-15 orthopaedic journal (defined by impact factor, Appendix) before ERAS submission. If publications could not be indexed in Scopus, PubMed was searched instead. The proportion of applicants with all listed ERAS publications successfully indexed was 88.1%. Research items reported on ERAS not verified through Scopus-indexed or PubMed-indexed citations were excluded from publication-based analyses.

Applicants were then matched to public Scopus profiles to assess post-ERAS research output. In a preliminary analysis, Scopus yielded more author profiles than Google Scholar. Applicants who could not be indexed on Scopus were excluded. Total present-day publication counts were obtained at the time of Scopus query and reflect publications occurring after ERAS application through residency, and approximately 10 years of practice. Total post-ERAS publications were tabulated by subtracting ERAS count from present-day count. This metric was derived because present-day publications, cumulative citations, and H-index all inherently include preapplication output in their calculation. Conversely, using post-ERAS publications allowed for independent assessment of associations between preresidency research and future output. Scopus data were queried from January 2010 through December 2025, allowing for 15 years of follow-up after the 2010 application cycle.

Several variables demonstrated right-skewed distributions with substantial variability and outliers. Therefore, associations between ERAS metrics and post-ERAS variables were evaluated using Spearman’s rank-order correlation. Association between total post-ERAS publication count and publication in a top-15 journal by the time of application was calculated with a one-way ANOVA test. Statistically significant correlations were defined as p < 0.05.

## Results

A total of 822 applicants applied to the authors’ orthopaedic surgery residency program in 2010. Of these, 513 were indexed on Scopus. The 309 applicants without Scopus records were excluded. Applicants averaged 2.87 ± 1.96 research experiences with 53.22 ± 57.67 hours per experience (Table I). The mean peer-reviewed output included 1.43 ± 2.80 journal articles/abstracts, 0.13 ± 0.96 book chapters, and 2.83 ± 4.71 poster/oral presentations. Applicants reported 5.52 ± 3.76 volunteer experiences (34.69 ± 47.79 hours/activity) and 2.75 ± 2.37 work experiences (69.06 ± 94.34 hours/job). The mean post-ERAS manuscript count was 17.63 ± 29.61. The large standard deviations relative to the means suggest a right-skewed distribution with a smaller subset of applicants contributing disproportionately higher publication counts.

**TABLE I:** Applicant Characteristics and Long-Term Publication Productivity

Variable	Mean ± SD
ERAS research characteristics	
Research experiences (n)	2.87 ± 1.96
Research hours per experience	53.22 ± 57.67
Peer-reviewed journal articles/abstracts (n)	1.43 ± 2.80
Peer-reviewed book chapters (n)	0.13 ± 0.96
Poster/oral presentations (n)	2.83 ± 4.71
Volunteer experiences	
Volunteer experiences (n)	5.52 ± 3.76
Volunteer hours per experience	34.69 ± 47.79
Work experiences	
Work experiences (n)	2.75 ± 2.37
Work hours per experience	69.06 ± 94.34
Long-term scholarly outcomes (from Scopus)	
Total publications (n)	18.69 ± 29.80
Post-ERAS publication count (n)	17.63 ± 29.61
Current citation count (n)	387.66 ± 683.81
Current h-index	7.15 ± 6.51

ERAS, Electronic Residency Application Service

**Fig. 1:**
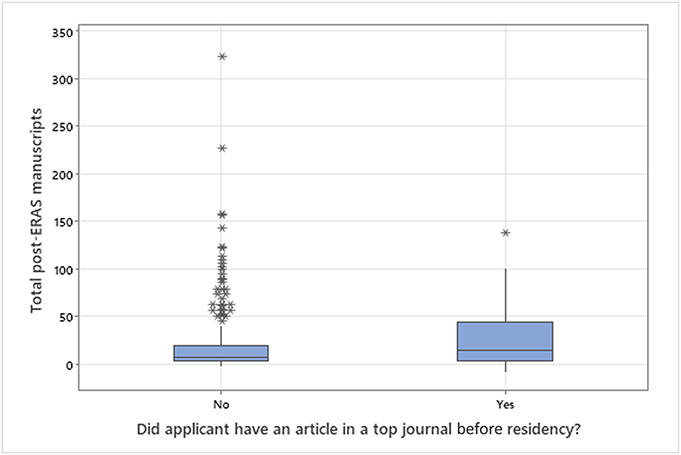
Association between Publishing in a top journal before residency and total post-ERAS manuscripts. There is a significant positive correlation between publishing in a top-15 orthopaedic journal before residency and publishing more academic papers in one’s career. ERAS = Electronic Residency Application Service.

Spearman rank coefficients showed statistically significant correlation between number of ERAS poster/oral presentations and subsequent post-ERAS manuscripts (P = 0.023; Table II and Fig. 1). However, no significant correlations were found between number of research experiences, hours of research experience, total research item count, number of manuscripts, and cumulative manuscript citations reported on ERAS and subsequent post-ERAS manuscripts. The number of volunteer and work experiences were not correlated with post-ERAS manuscripts. One-way ANOVA demonstrated that applicants with at least 1 top-15 orthopaedic journal publication on application produced significantly more post-ERAS manuscripts than those without such publications (26.6 ± 32.8 vs. 16.7 ± 29.1; F(1,510) = 4.93, p = 0.027; η^2^ = 0.01).

**TABLE II:** Associations Between ERAS Application Variables and Post-ERAS Manuscripts Based on Spearman Rank Coefficients

ERAS Variable	P
Poster/oral presentations	**0.023**
Number of research experiences	0.129
Hours of research experience	0.501
Total research item count	0.571
Number of manuscripts	0.713
Cumulative manuscript citations	0.308

ERAS = Electronic Residency Application Service.

## Discussion

This study found that qualitative markers of research engagement, specifically conference presentations and caliber of publications, were associated with long-term research output, whereas quantity of research items was not. Applicants who presented their work (posters/oral presentations) produced significantly more publications following residency application, reflecting increased early-career publication output. However, total number of research items and manuscripts listed on ERAS application, as well as cumulative citations of those works, demonstrated no significant correlation with subsequent publication output. In addition, publication in a high-impact orthopaedic journal was significantly positively correlated with future research output, underscoring the importance of publication quality. These findings suggest presenting at conferences or publishing in respected journals better reflect meaningful scholarly engagement than research volume alone in this cohort.

These results align with several studies similarly challenging the assumption that research quantity is correlated with later academic output in orthopaedic surgery^12-14^. A previous study by Su et al. concluded there was no correlation between the number of publications listed on an ERAS application and performance in orthopaedic surgery residency^12^. Similarly, Goss et al. reported that publications from medical school, residency, or fellowship were not associated with research productivity in the first decade of practice^13^. In addition, Levy et al. determined that research productivity on fellowship applications did not predict performance during fellowship training^14^.

By contrast, other studies found that research productivity during training correlates with greater subsequent scholarly activity^7,15^. Donley et al. reported that having 2 or more publications before residency correlates with increased publications in residency^7^. However, their study omitted career research productivity or variables such as publication count, top journal publication, or nonmanuscript research items. Similarly, Rompala et al. found publication output throughout medical training was associated with increased academic productivity as an attending^15^. However, their cohort only included surgeons in academic medicine, not accounting for the 57% of residents who leave academia, thus introducing selection bias^16^. Our study examined an entire cohort of orthopaedic surgery residency applicants across all future employment settings.

The heterogeneous results of prior literature highlight difficulties in determining which preresidency research variables are meaningfully associated with long-term research output. Some inconsistency stems from variation in specific study questions and parameters. For instance, several studies evaluated research productivity using career-long metrics; however, these measures inherently include manuscripts produced before residency, meaning the predictor and outcome are not independent. This overlap complicates determining whether early research involvement drives later scholarly activity or simply appears to because the same publications were counted twice. For example, both Goss et al. and Donley et al. used h-index and total career publications—metrics that incorporate preresidency work^7,13^. By contrast, our isolation of post-ERAS publications enables more accurate assessment of independent publication productivity after residency application. However, the timing of post-ERAS publications cannot be fully clarified in our study. Publications after ERAS application may represent work initiated before residency that reached publication later. Therefore, the observed associations may partially reflect continuity of involvement rather than new scholarly activity.

While research quantity is easy to track and compare across applicants, our data show that qualitative measures such as publishing in a top journal or conference presentation are associated with future research productivity rather than quantity of projects completed. These findings may be explained by the fact that publishing in top journals requires greater quality research, likely with greater time commitment, increased mentorship, and superior demonstrated ability. Acceptance at scientific meetings and publication in higher-impact journals may reflect success in more rigorous peer review, serving as a proxy for depth of engagement, project completion, and dissemination^17^.

Furthermore, conference presentations require project ownership and time commitment. For residency programs, this distinction matters: Relying solely on research quantity may inadvertently reward superficial involvement, particularly as research inflation continues to rise. Likewise, applicants may benefit from investing deeply into fewer, more substantial projects than from accumulating long research lists. Therefore, our findings support shifting toward evaluating the quality and context of an applicant’s scholarly work instead of numerical research totals.

This study has several limitations. First, the cohort was composed of applicants from a single US residency orthopaedic surgery program and selection priorities vary across programs^18^. This program ranks in the top 40 in research output, and thus, applicants may average more research experience than the median candidate^11^. That said, this cohort constitutes over half of the national orthopaedic surgery application class in 2010 and national data are not available on this topic^19^. Second, this study relied on Scopus to index publications from residency until 10 years into subjects’ careers. While Scopus indexes from over 7,000 publishers, it does not include every published article^20^. In addition, not all applicants had Scopus profiles, introducing potential selection bias, as individuals with Scopus profiles more likely have active research involvement^21^. This would likely bias the sample toward those who prioritized research in their careers and potentially overstate the relationship between preresidency and subsequent research output. However, we found that research productivity on ERAS applications was not correlated with post-ERAS manuscripts.

This study was also unable to assess the depth of individual contributions to research projects. In addition, applicants with advanced degrees were not analyzed separately. These individuals may have had prior nonorthopaedic research output captured in total publication counts but not reflected in field-specific metrics such as publication in top orthopaedic journals. Furthermore, the application process and expectations have evolved since the 2010 class; however, it was necessary to analyze applicants from this period to observe their productivity into their careers. Finally, this study relies on self-reported ERAS information, with potential variability in applicant reporting.

This interpretation should be considered within the context of our study design. While the predictors examined are aspects of research quality, our outcome of post-ERAS publication count is a quantitative metric. Therefore, these results do not assess the quality of future scholarly work, but rather the volume. Moreover, research productivity is only 1 proxy of a successful career as an orthopaedic surgeon. Our findings should not be interpreted as supporting increased reliance on research metrics in residency selection. Rather, they suggest that commonly used measures may not be meaningfully associated with future publication output. An overemphasis on research quantity may misrepresent applicants’ potential and overlook other attributes critical to the development of skilled and compassionate physicians.^22^

Future research could build on this study by expanding to multi-institutional cohorts to improve generalizability. In addition, this cohort could be followed further into their careers to determine if these associations persist. Further analysis could segregate post-ERAS manuscripts by which were produced during residency or as a fellow or attending physician. Finally, future studies should examine the role of socioeconomic class in research opportunity, given the high cost of research years.^23^

## Conclusion

Markers of substantive research engagement—such as conference presentation and publication in higher-impact orthopaedic journals—were associated with increased future research output, whereas total research items, manuscript count, and preresidency citations were not. These findings suggest that the depth and rigor of preresidency scholarship may be more strongly associated with future publication output than research volume alone. Greater emphasis on the quality of scholarly contributions may improve assessment of academic potential, and applicants may benefit from prioritizing meaningful involvement versus superficial participation. Importantly, research output is only 1 measure of success, and orthopaedic surgery residency programs should prioritize developing skilled and compassionate providers.

## Appendix

Supporting material provided by the authors is posted with the online version of this article as a data supplement at jbjs.org (http://links.lww.com/JBJSOA/B266). This content was not copy edited or verified by JBJS.

## References

[1] Kheir MM Tan TL Rondon AJ Chen AF. The fate of unmatched orthopaedic applicants: risk factors and outcomes. JBJS Open Access. 2020;5(2):e20.00043. 10.2106/JBJS.OA.20.00043 PMC7418921 32832827

[2] Lin EH Feingold CL Jagasia AA Tercyak SC Agarwalla A Liu JN. Effect of the USMLE step 1 grading change on orthopedic surgery residency applications: national resident matching program charting outcomes from 2011 to 2024. Orthopedics. 2025;48(3):e139-46. 40272028 10.3928/01477447-20250414-01

[3] Varieur BM White RC Bono CM. Increasing research productivity and step 2 score among matched orthopaedic surgery residents: a forecasting analysis to 2040. J Am Acad Orthop Surg. 2025;33(10):548-53. 40036697 10.5435/JAAOS-D-24-01106

[4] DePasse JM Palumbo MA Eberson CP Daniels AH. Academic characteristics of orthopaedic surgery residency applicants from 2007 to 2014. J Bone Joint Surg Am. 2016;98(9):788-95. 27147692 10.2106/JBJS.15.00222

[5] White RC Vutukuri R Saraf SM Rumps MV Mulcahey MK. The value of research in the orthopaedic surgery residency applicant: a pilot survey of orthopaedic surgery residency program directors. JBJS Open Access. 2025;10(2):e24.00216. 10.2106/JBJS.OA.24.00216 PMC12080675 40406035

[6] Lemme NJ Li NY Twomey-Kozak J DeFroda SF Silber Z Daniels AH Eberson CP. Characterization and fate of unpublished research articles reported by orthopedic surgery residency applicants. J Surg Edu. 2020;77(3):698-703. 10.1016/j.jsurg.2019.11.005 31852587

[7] Donley C McCrosson M Prahad S Campbell C Zhao F Amireddy N Johnson M. High research productivity during orthopaedic surgery residency may be predicted by number of publications as a medical student. JB JS Open Access. 2024;9(1):e23.00105. 10.2106/JBJS.OA.23.00105 PMC10817159 38293278

[8] Acevedo D Destiné H Murdock CJ LaPorte D Aiyer AA. Correlation between research productivity during and after orthopaedic surgery training. Surg Open Sci. 2024;18:98-102. 38440317 10.1016/j.sopen.2024.02.010 PMC10910153

[9] Banko L Riesenburger N Patel RV Gilligan C Cosgrove GR Chiocca EA Proctor MR Patel AJ Bi WL. Predictive value of neurosurgery applicant metrics on resident academic productivity. Neurosurgery. 2025;96(6):1206-16. 39526786 10.1227/neu.0000000000003251

[10] Alsoof D Balmaceno-Criss M Kovoor M Casey J Johnson K McDonald CL Diebo BG Kuris EO Daniels AH. Does research training lead to academic success in orthopedic surgery? An analysis of U.S academic orthopedic surgeons. Orthop Rev. 2022;14(4):38655. 10.52965/001c.38655 PMC9560893 36263194

[11] Doximity Residency Navigator 2025-2026. Doximity. Accessed April 18, 2026.

[12] Su CA Furdock RJ Rascoe AS Vallier HA Liu RW Voos JE Gillespie RJ. Which application factors are associated with outstanding performance in orthopaedic surgery residency? Clin Orthop Relat Res. 2023;481(2):387-96. 36083836 10.1097/CORR.0000000000002373 PMC9831202

[13] Goss ML McNutt S Bible JE. Does publication history predict future publication output in orthopaedics? Cureus. 2021;13(5):e15273. 34194877 10.7759/cureus.15273 PMC8234811

[14] Levy HA Boere P Randell Z Bodnar J Paulik J Spina NT Spiker WR Lawrence BD Brodke DS Kurd MF Rihn JA Canseco JA Schroeder GD Kepler CK Vaccaro AR Currier B Huddleston PM Nassr AN Freedman BA Sebastian AS Hilibrand AS Karamian BA. Factors related to clinical performance in spine surgery fellowship: can we predict success. J Am Acad Orthop Surg. 2024;32(18):e940-50. 39008910 10.5435/JAAOS-D-24-00120

[15] Rompala A Sudah SY Miller AS Gaccione AG Nicholson AD Namdari S Menendez ME. Predicting academic productivity among American shoulder and elbow surgeons fellowship faculty from publications acquired before and during surgical training. J Shoulder Elbow Surg. 2024;33(10):e523-28. 38582253 10.1016/j.jse.2024.02.025

[16] AAOS. Orthopaedic Practice in the U.S. 2018. (AAOS: 2019). AAOS Department of Clinical Quality and Value; 24-30; https://www.aaos.org/globalassets/quality-and-practice-resources/census/2018-census.pdf (2019, 2026).

[17] Tumin D Tobias JD. The peer review process. Saudi J Anaesth. 2019;13(suppl 1):S52-8. 30930722 10.4103/sja.SJA_544_18 PMC6398293

[18] Meade PJ Amin SJ Sheena J Stamm MA Mulcahey MK. Doximity orthopaedic surgery program rankings are associated with academic productivity. JBJS Open Access. 2023;8(1):e22.00081 10.2106/JBJS.OA.22.00081 PMC9820772 36698990

[19] Pinpin C White PB Nellans KW Bitterman AD Mulcahey MK Cohn RM. Exponential growth in female residency applicants in orthopaedic surgery over the past 15 years. JB JS Open Access. 2023;8(2):e23.00004 10.2106/JBJS.OA.23.00004 PMC10226613 37255671

[20] Scopus Content. Elsevier; https://www.elsevier.com/products/scopus/content (2026).

[21] Alter N Daiem M Pontell ME Galdyn I Golinko M Perdikis G Lineaweaver W. Limitations of academic bibliometric indices: the need for more comprehensive metrics. Ann Plast Surg. 2025;95(6):603-6. 41263888 10.1097/SAP.0000000000004484

[22] Jernigan MA Carbonneau KJ. Advancing health professions education: a review of holistic admissions and competency-based admissions practices. Med Educ Online. 2025;30(1):2486979. 40183673 10.1080/10872981.2025.2486979 PMC11980201

[23] Jacobsen A Kabbur G Freese RL Rypka KJ Goldfarb N. Socioeconomic factors and financial burdens of research “gap years” for dermatology residency applicants. Int J Womens Dermatol. 2023;9(3):e099. 37547567 10.1097/JW9.0000000000000099 PMC10403007

